# Tetra­kis(5,7-dimethyl­quinolin-8-olato-κ^2^ *N*,*O*)zirconium(IV) dimethyl­form­amide disolvate

**DOI:** 10.1107/S1600536812042092

**Published:** 2012-10-13

**Authors:** Maryke Steyn, Hendrik G. Visser, Andreas Roodt

**Affiliations:** a Department of Chemistry, University of the Free State, PO Box 339, Bloemfontein 9300, South Africa

## Abstract

In the title compound, [Zr(C_11_H_10_NO)_4_]·2C_3_H_7_NO, the Zr^IV^ ion is coordinated by four bidentate 5,7-dimethylquinolin-8-olate ligands in a slightly distorted square-anti­prismatic coordination environment. The asymmetric unit also contains two *N*,*N*′-dimethyl­formamide (DMF) solvent mol­ecules. In the crystal, a weak C—H⋯O hydrogen bond links the complex mol­ecule to a solvent mol­ecule and weak π–π stacking inter­actions [centroid–centroid distance = 3.671 (3) Å] also occur. One of the DMF solvent mol­ecules was refined as disordered over three sets of sites, with refined occupancies in the ratio of 0.391 (9):0.342 (10):0.267 (7).

## Related literature

For *N*,*O*- and *O*,*O*′-bidentate ligand complexes of zirconium and hafnium, see: Calderazzo *et al.* (1998 ▶); Demakopoulos *et al.* (1995 ▶); Steyn *et al.* (2008 ▶, 2011 ▶); Viljoen *et al.* (2008 ▶, 2009*a* ▶,*b* ▶; 2010*a* ▶,*b* ▶); Zherikova *et al.* (2005 ▶, 2006 ▶, 2008 ▶). For our ongoing research of structure reactivity relationships in catalysis, separation chemistry and other industrial reaction mechanisms, see: Roodt *et al.* (2011 ▶); Schutte *et al.* (2011 ▶); Brink *et al.* (2010 ▶); Ferreira *et al.* (2007 ▶); Haumann *et al.* (2004 ▶). 
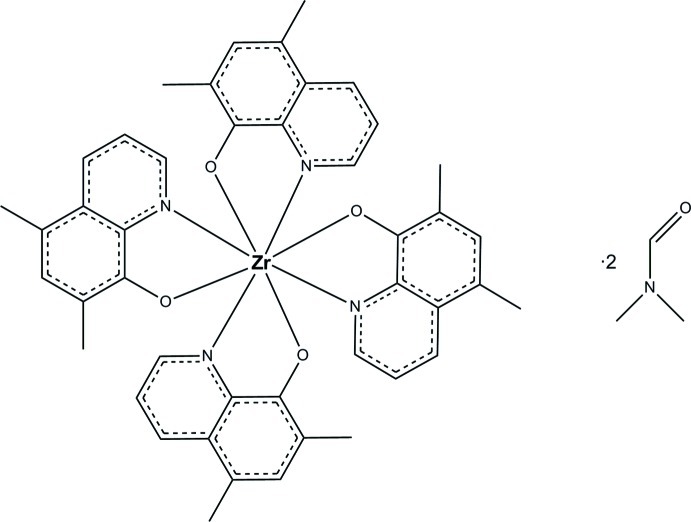


## Experimental

### 

#### Crystal data

[Zr(C_11_H_10_NO)_4_]·2C_3_H_7_NO
*M* *_r_* = 926.21
Orthorhombic, 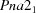

*a* = 15.572 (5) Å
*b* = 18.706 (5) Å
*c* = 15.853 (5) Å
*V* = 4618 (2) Å^3^
*Z* = 4
Mo *K*α radiation
μ = 0.29 mm^−1^
*T* = 100 K
0.26 × 0.14 × 0.13 mm

#### Data collection

Bruker APEXII CCD diffractometer
Absorption correction: multi-scan (*SADABS*; Bruker, 2004 ▶) *T*_min_ = 0.928, *T*_max_ = 0.963
61276 measured reflections
11142 independent reflections
8497 reflections with *I* > 2σ(*I*)
*R*_int_ = 0.064

#### Refinement

*R*[*F* ^2^ > 2σ(*F* ^2^)] = 0.041
*wR*(*F* ^2^) = 0.099
*S* = 1.02
11142 reflections
671 parameters
299 restraints
H-atom parameters constrained
Δρ_max_ = 0.42 e Å^−3^
Δρ_min_ = −0.39 e Å^−3^
Absolute structure: Flack (1983 ▶), 5375 Friedel pairs
Flack parameter: −0.01 (3)

### 

Data collection: *APEX2* (Bruker, 2005 ▶); cell refinement: *SAINT* (Bruker, 2004 ▶); data reduction: *SAINT*; program(s) used to solve structure: *SHELXS97* (Sheldrick, 2008 ▶); program(s) used to refine structure: *SHELXL97* (Sheldrick, 2008 ▶); molecular graphics: *DIAMOND* (Brandenburg & Putz, 2005 ▶); software used to prepare material for publication: *WinGX* (Farrugia, 1999 ▶).

## Supplementary Material

Crystal structure: contains datablock(s) global, I. DOI: 10.1107/S1600536812042092/lh5537sup1.cif

Structure factors: contains datablock(s) I. DOI: 10.1107/S1600536812042092/lh5537Isup2.hkl

Additional supplementary materials:  crystallographic information; 3D view; checkCIF report

## Figures and Tables

**Table 1:** Hydrogen-bond geometry (Å, °)

*D*—H⋯*A*	*D*—H	H⋯*A*	*D*⋯*A*	*D*—H⋯*A*
C14*A*—H14*E*⋯O201^i^	0.96	2.43	3.358 (7)	161

Symmetry code: (i) 
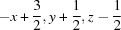
.

**Fig. 1.**
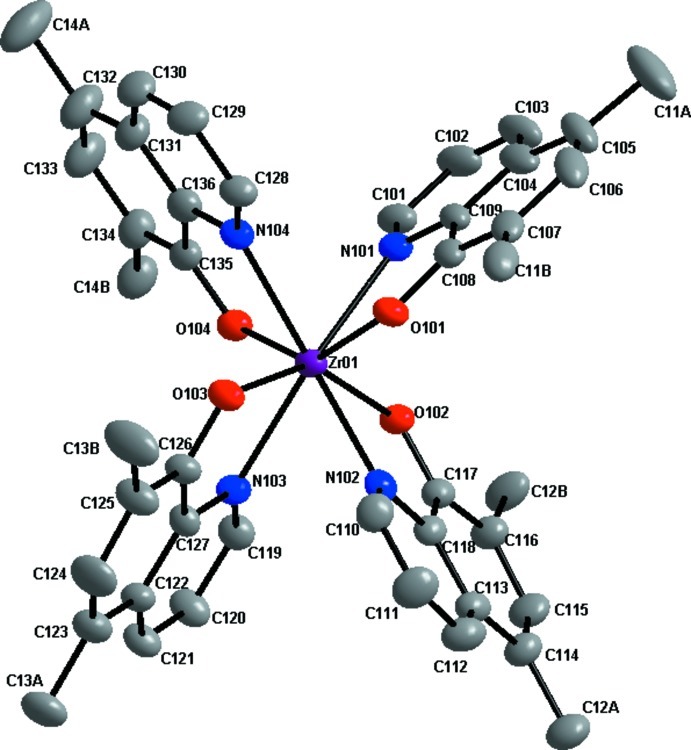
The molecular structre of the Zr complex of the title compound. Displacement ellipsoids are drawn at the 50% probability level. H atoms and the solvent molecules are omitted for clarity.

**Fig. 2.**
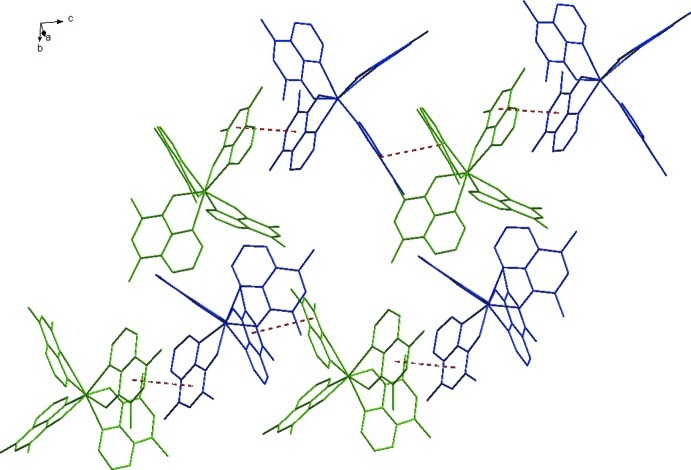
Part of the crystal structure with weak π–π interactions shown as dashed lines.

## supplementary crystallographic information

### Comment

This study forms part of our ongoing research of structure reactivity relationships in catalysis, separation chemistry and other industrial reaction mechanisms including radio pharmacy (Roodt *et al.* 2011; Schutte *et al.* 2011; Brink *et al.* 2010; Ferreira *et al.* 2007; Haumann *et al.* 2004; Steyn *et al.* 2008, 2011; Viljoen *et al.* 2008, 2009*a*,*b*, 2010*a*,*b*).

The asymmetric unit of the title compound, [Zr(C_10_H_11_NO)_4_].2C_3_H_7_NO, with C_10_H_11_NO (diMeOx) = 5,7-Dimethyl-8-quinolinol, consists of a Zr^IV^ ion coordinated to four bidentate ligands (diMeOx), as well as two *N*,*N*'-dimethylformamide (DMF) solvent molecules. In the complex molecule (Fig. 1) the Zr^IV^ ion lies at the centre of an approximate square antiprismatic coordination polyhedron of the N,*O*-coordination ligand atoms, with a small distortion towards dodecahedral geometry. The Zr—N and Zr—O bond distances range from 2.094 (2) to 2.117 (2) Å and 2.398 (2) to 2.438 (2) Å, respectively. The N—Zr—O bite angles range from 69.70 (8)° to 70.55 (8)°.

In the crystal, a weak C—H···O hydrogen bond connects the complex molecule to a solvent molecule (Table 1). In addition, weak π–π interactions exist between the pyridine rings of the diMeOx ligand and symmetry related molecules (1 - *x*, 1 - *y*, 1/2 + *z*) , with interplanar and centroid-to-centroid distances of 3.433 (4) Å and 3.671 (3) Å, respectively (Figure 2).

### Experimental

Chemicals were purchased from Sigma-Aldrich and used as received. ZrCl_4_ (101.3 mg, 0.435 mmol) and 5,7-Dimethyl-8-quinolinol (diMeOxH) (228.2 mg, 1.317 mmol) was separately dissolved in DMF (2.5 ml ea) and heated to 60°C. The diMeOxH solution as added drop-wise to the zirconium solution and stirred at 333 K for 30 minutes. The reaction solution was removed from heating, covered and left to stand. Red cuboid crystals, suitable for single X-Ray diffraction, formed after 10 days. (Yield: 203 mg, 79%).

### Refinement

H atoms were placed inidealized positions (C—H = 0.93–0.96Å) and constrained to ride on their parent atoms with *U*_iso_(H) = 1.2–1.5*U*_eq_(C). The highest residual electron density was located 0.95 Å from O102. One of the DMF solvent molecules was refined as disordered over three sets of sites with refined occupancies in a ratio of 0.391 (9):0.342 (10):0.267 (7).

### Crystal data

**Table:**

[Zr(C_11_H_10_NO)_4_]·2C_3_H_7_NO	*F*(000) = 1936
*M**_r_* = 926.21	*D*_x_ = 1.332 Mg m^−^^3^
Orthorhombic, *P**n**a*2_1_	Mo *K*α radiation, λ = 0.71073 Å
Hall symbol: P 2c -2n	Cell parameters from 9914 reflections
*a* = 15.572 (5) Å	θ = 2.6–24.6°
*b* = 18.706 (5) Å	µ = 0.29 mm^−^^1^
*c* = 15.853 (5) Å	*T* = 100 K
*V* = 4618 (2) Å^3^	Cuboid, red
*Z* = 4	0.26 × 0.14 × 0.13 mm

### Data collection

**Table:**

Bruker APEXII CCD diffractometer	11142 independent reflections
Radiation source: sealed tube	8497 reflections with *I* > 2σ(*I*)
Graphite monochromator	*R*_int_ = 0.064
φ and ω scans	θ_max_ = 28°, θ_min_ = 2.1°
Absorption correction: multi-scan (*SADABS*; Bruker, 2004)	*h* = −20→20
*T*_min_ = 0.928, *T*_max_ = 0.963	*k* = −22→24
61276 measured reflections	*l* = −20→20

### Refinement

**Table:**

Refinement on *F*^2^	Secondary atom site location: difference Fourier map
Least-squares matrix: full	Hydrogen site location: inferred from neighbouring sites
*R*[*F*^2^ > 2σ(*F*^2^)] = 0.041	H-atom parameters constrained
*wR*(*F*^2^) = 0.099	*w* = 1/[σ^2^(*F*_o_^2^) + (0.0485*P*)^2^ + 0.3005*P*] where *P* = (*F*_o_^2^ + 2*F*_c_^2^)/3
*S* = 1.02	(Δ/σ)_max_ = 0.002
11142 reflections	Δρ_max_ = 0.42 e Å^−^^3^
671 parameters	Δρ_min_ = −0.39 e Å^−^^3^
299 restraints	Absolute structure: Flack (1983), 5375 Friedel pairs
Primary atom site location: structure-invariant direct methods	Flack parameter: −0.01 (3)

### Special details

**Table:**

Geometry. All s.u.'s (except the s.u. in the dihedral angle between two l.s. planes) are estimated using the full covariance matrix. The cell s.u.'s are taken into account individually in the estimation of s.u.'s in distances, angles and torsion angles; correlations between s.u.'s in cell parameters are only used when they are defined by crystal symmetry. An approximate (isotropic) treatment of cell s.u.'s is used for estimating s.u.'s involving l.s. planes.
Refinement. Refinement of *F*^2^ against ALL reflections. The weighted *R*-factor *wR* and goodness of fit *S* are based on *F*^2^, conventional *R*-factors *R* are based on *F*, with *F* set to zero for negative *F*^2^. The threshold expression of *F*^2^ > 2σ(*F*^2^) is used only for calculating *R*-factors(gt) *etc*. and is not relevant to the choice of reflections for refinement. *R*-factors based on *F*^2^ are statistically about twice as large as those based on *F*, and *R*- factors based on ALL data will be even larger.

### Fractional atomic coordinates and isotropic or equivalent isotropic displacement parameters (Å^2^)

**Table:**

	*x*	*y*	*z*	*U*_iso_*/*U*_eq_	Occ. (<1)
Zr01	0.966490 (14)	0.144904 (13)	0.35471 (2)	0.02304 (7)	
N102	0.96042 (15)	0.03648 (14)	0.43981 (17)	0.0262 (6)	
O101	0.89103 (13)	0.17184 (11)	0.46024 (13)	0.0297 (5)	
O104	1.02836 (11)	0.19244 (11)	0.24980 (13)	0.0271 (5)	
N104	0.87759 (15)	0.23775 (13)	0.29748 (17)	0.0262 (6)	
O103	0.85773 (12)	0.09319 (11)	0.30675 (14)	0.0279 (5)	
N101	1.01720 (16)	0.25663 (13)	0.41574 (17)	0.0294 (6)	
N103	1.01035 (15)	0.04558 (13)	0.26899 (16)	0.0251 (6)	
O102	1.08748 (12)	0.12297 (11)	0.40715 (14)	0.0271 (5)	
C116	1.1890 (2)	0.04673 (17)	0.4780 (2)	0.0326 (7)	
C128	0.79960 (18)	0.25709 (16)	0.3227 (2)	0.0295 (7)	
H128	0.7761	0.2349	0.3698	0.035*	
C110	0.8931 (2)	−0.00201 (18)	0.4599 (2)	0.0339 (8)	
H110	0.8392	0.0135	0.4427	0.041*	
C122	0.95422 (19)	−0.05997 (17)	0.1978 (2)	0.0260 (7)	
C107	0.8621 (2)	0.23826 (18)	0.5857 (2)	0.0352 (8)	
C125	0.79013 (19)	−0.00990 (18)	0.2458 (2)	0.0363 (8)	
C121	1.03962 (19)	−0.07799 (19)	0.1757 (2)	0.0320 (8)	
H121	1.0504	−0.1193	0.1448	0.038*	
C118	1.03964 (18)	0.01552 (17)	0.46759 (19)	0.0247 (6)	
C111	0.8992 (2)	−0.0659 (2)	0.5063 (2)	0.0423 (9)	
H111	0.8502	−0.0922	0.519	0.051*	
C109	0.97617 (19)	0.27293 (17)	0.4900 (2)	0.0291 (7)	
C120	1.1060 (2)	−0.03520 (18)	0.1995 (2)	0.0352 (8)	
H120	1.1621	−0.0464	0.1841	0.042*	
C114	1.1373 (2)	−0.06654 (18)	0.5408 (2)	0.0332 (8)	
C117	1.10771 (19)	0.06285 (17)	0.4490 (2)	0.0278 (7)	
C129	0.75120 (19)	0.30983 (17)	0.2808 (2)	0.0348 (8)	
H129	0.6965	0.3216	0.2997	0.042*	
C119	1.0886 (2)	0.02635 (18)	0.2479 (2)	0.0307 (7)	
H119	1.1344	0.0545	0.2657	0.037*	
C134	1.03493 (19)	0.27832 (19)	0.1369 (2)	0.0355 (8)	
C127	0.94307 (19)	0.00394 (16)	0.2438 (2)	0.0251 (7)	
C135	0.9944 (2)	0.24636 (17)	0.2046 (2)	0.0270 (7)	
C131	0.8681 (2)	0.32476 (19)	0.1829 (2)	0.0343 (7)	
C112	0.9788 (2)	−0.0889 (2)	0.5328 (2)	0.0389 (9)	
H112	0.9837	−0.1313	0.563	0.047*	
C115	1.2008 (2)	−0.01833 (18)	0.5218 (2)	0.0381 (8)	
H115	1.2562	−0.0294	0.5391	0.046*	
C101	1.0838 (2)	0.29617 (16)	0.3917 (2)	0.0341 (8)	
H101	1.1107	0.2859	0.3407	0.041*	
C113	1.0527 (2)	−0.04862 (17)	0.5143 (2)	0.0296 (7)	
C102	1.1149 (2)	0.35297 (18)	0.4411 (3)	0.0429 (9)	
H102	1.1621	0.3794	0.4231	0.051*	
C104	1.0026 (2)	0.32912 (19)	0.5431 (3)	0.0400 (9)	
C136	0.91150 (19)	0.27068 (17)	0.2282 (2)	0.0294 (7)	
C14B	1.1235 (2)	0.2566 (2)	0.1106 (2)	0.0412 (9)	
H14A	1.165	0.2818	0.1441	0.062*	
H14B	1.1318	0.268	0.0521	0.062*	
H14C	1.1304	0.2061	0.1187	0.062*	
C133	0.9898 (3)	0.3325 (2)	0.0928 (3)	0.0465 (10)	
H133	1.0169	0.3534	0.0467	0.056*	
C126	0.86090 (18)	0.03010 (16)	0.2668 (2)	0.0272 (7)	
C12A	1.1540 (2)	−0.13471 (18)	0.5890 (2)	0.0427 (9)	
H12A	1.2146	−0.1402	0.5978	0.064*	
H12B	1.1327	−0.1747	0.5572	0.064*	
H12C	1.1252	−0.1327	0.6425	0.064*	
C124	0.8028 (2)	−0.07534 (18)	0.2024 (2)	0.0428 (9)	
H124	0.7545	−0.1024	0.1897	0.051*	
C108	0.9078 (2)	0.22615 (17)	0.5113 (2)	0.0302 (7)	
C103	1.0751 (2)	0.36913 (19)	0.5161 (3)	0.0460 (10)	
H103	1.0957	0.4064	0.5492	0.055*	
C13A	0.8899 (2)	−0.17105 (18)	0.1312 (3)	0.0425 (9)	
H13A	0.8342	−0.1914	0.1216	0.064*	
H13B	0.924	−0.2035	0.164	0.064*	
H13C	0.9176	−0.1625	0.078	0.064*	
C106	0.8905 (2)	0.2956 (2)	0.6375 (2)	0.0469 (10)	
H106	0.8612	0.303	0.6878	0.056*	
C11B	0.7879 (2)	0.1918 (2)	0.6073 (2)	0.0416 (9)	
H11A	0.7388	0.206	0.5747	0.062*	
H11B	0.775	0.1966	0.6663	0.062*	
H11C	0.8018	0.143	0.595	0.062*	
C105	0.9568 (3)	0.3407 (2)	0.6200 (3)	0.0506 (12)	
C13B	0.7013 (2)	0.0169 (2)	0.2666 (3)	0.0574 (12)	
H13D	0.6854	0.001	0.322	0.086*	
H13E	0.6611	−0.0014	0.2261	0.086*	
H13F	0.7008	0.0682	0.2649	0.086*	
C12B	1.2621 (2)	0.09893 (19)	0.4674 (3)	0.0428 (9)	
H12D	1.2882	0.092	0.4131	0.064*	
H12E	1.304	0.091	0.5108	0.064*	
H12F	1.2406	0.1469	0.4714	0.064*	
C14A	0.8651 (3)	0.4165 (3)	0.0639 (3)	0.0684 (14)	
H14D	0.9045	0.4356	0.0231	0.103*	
H14E	0.848	0.4536	0.1022	0.103*	
H14F	0.8154	0.398	0.0356	0.103*	
C130	0.7850 (2)	0.34339 (18)	0.2126 (2)	0.0377 (8)	
H130	0.7536	0.3787	0.1852	0.045*	
C123	0.8805 (2)	−0.10150 (17)	0.1781 (2)	0.0328 (8)	
C132	0.9089 (3)	0.3563 (2)	0.1132 (3)	0.0496 (10)	
C11A	0.9852 (3)	0.3981 (3)	0.6804 (3)	0.0790 (17)	
H11D	0.976	0.4442	0.6553	0.119*	
H11E	1.0451	0.3922	0.6929	0.119*	
H11F	0.9525	0.3946	0.7316	0.119*	
N201	0.5982 (2)	0.0128 (2)	0.8446 (3)	0.0738 (11)	
C202	0.5442 (3)	−0.0454 (3)	0.8137 (3)	0.0685 (13)	
H20A	0.5562	−0.0537	0.7551	0.103*	
H20B	0.4848	−0.0326	0.8202	0.103*	
H20C	0.5559	−0.088	0.8453	0.103*	
C203	0.5743 (5)	0.0405 (5)	0.9247 (5)	0.144 (3)	
H20D	0.6092	0.0813	0.9377	0.216*	
H20E	0.5824	0.0045	0.9671	0.216*	
H20F	0.515	0.0545	0.9235	0.216*	
C201	0.6631 (3)	0.0338 (3)	0.7986 (5)	0.0925 (18)	
H201	0.696	0.0695	0.8237	0.111*	
O201	0.6878 (2)	0.0165 (2)	0.7320 (3)	0.0958 (13)	
N31	0.372 (2)	0.2574 (12)	0.8644 (9)	0.142 (6)	0.342 (10)
C31A	0.464 (2)	0.2579 (16)	0.8591 (10)	0.141 (8)	0.342 (10)
H31A	0.485	0.2097	0.8564	0.211*	0.342 (10)
H31B	0.4875	0.2811	0.908	0.211*	0.342 (10)
H31C	0.4815	0.2833	0.8093	0.211*	0.342 (10)
C31B	0.331 (2)	0.3202 (14)	0.8686 (13)	0.158 (9)	0.342 (10)
H31D	0.3721	0.3585	0.8649	0.237*	0.342 (10)
H31E	0.3009	0.3234	0.9212	0.237*	0.342 (10)
H31F	0.2911	0.3237	0.8228	0.237*	0.342 (10)
C31C	0.3383 (19)	0.1890 (12)	0.8670 (13)	0.151 (6)	0.342 (10)
H31J	0.2808	0.1767	0.874	0.181*	0.342 (10)
O31	0.4022 (14)	0.1459 (8)	0.8578 (11)	0.169 (7)	0.342 (10)
N32	0.5527 (18)	0.2747 (12)	0.8555 (18)	0.163 (8)	0.267 (7)
C32B	0.476 (2)	0.3148 (16)	0.848 (3)	0.171 (10)	0.267 (7)
H32A	0.489	0.3627	0.8307	0.257*	0.267 (7)
H32B	0.4464	0.3157	0.9011	0.257*	0.267 (7)
H32C	0.4392	0.2929	0.8062	0.257*	0.267 (7)
C32C	0.546 (2)	0.2060 (11)	0.8783 (12)	0.169 (10)	0.267 (7)
H32D	0.4869	0.1931	0.8826	0.253*	0.267 (7)
H32E	0.5738	0.1992	0.932	0.253*	0.267 (7)
H32F	0.5738	0.1765	0.8368	0.253*	0.267 (7)
C32A	0.6322 (19)	0.3019 (16)	0.841 (2)	0.198 (11)	0.267 (7)
H32J	0.6379	0.3497	0.8257	0.238*	0.267 (7)
O32	0.6989 (19)	0.2633 (15)	0.8475 (15)	0.237 (12)	0.267 (7)
N33	0.3685 (14)	0.2750 (9)	0.8788 (12)	0.127 (5)	0.391 (9)
C33A	0.4579 (15)	0.2793 (11)	0.8624 (16)	0.112 (6)	0.391 (9)
H33A	0.4857	0.2372	0.8836	0.167*	0.391 (9)
H33B	0.4811	0.3207	0.8899	0.167*	0.391 (9)
H33C	0.4672	0.2829	0.8027	0.167*	0.391 (9)
C33C	0.3514 (15)	0.2052 (7)	0.9044 (9)	0.122 (6)	0.391 (9)
H33D	0.4044	0.1812	0.9166	0.183*	0.391 (9)
H33E	0.3217	0.1803	0.8603	0.183*	0.391 (9)
H33F	0.3163	0.2061	0.9542	0.183*	0.391 (9)
C33B	0.3106 (17)	0.3250 (11)	0.8571 (17)	0.130 (6)	0.391 (9)
H33J	0.3285	0.3643	0.8258	0.156*	0.391 (9)
O33	0.2314 (12)	0.3201 (7)	0.8785 (8)	0.142 (6)	0.391 (9)

### Atomic displacement parameters (Å^2^)

**Table:**

	*U*^11^	*U*^22^	*U*^33^	*U*^12^	*U*^13^	*U*^23^
Zr01	0.01669 (11)	0.02212 (12)	0.03031 (13)	0.00031 (10)	−0.00125 (16)	−0.00248 (17)
N102	0.0214 (13)	0.0287 (15)	0.0285 (15)	0.0003 (11)	0.0024 (11)	−0.0051 (12)
O101	0.0275 (11)	0.0256 (11)	0.0360 (13)	0.0039 (9)	0.0014 (9)	−0.0050 (10)
O104	0.0177 (10)	0.0272 (11)	0.0365 (12)	−0.0001 (8)	−0.0009 (9)	−0.0015 (10)
N104	0.0174 (11)	0.0264 (13)	0.0347 (15)	0.0011 (10)	−0.0020 (10)	−0.0047 (12)
O103	0.0164 (9)	0.0280 (11)	0.0393 (12)	0.0005 (8)	−0.0008 (9)	−0.0068 (10)
N101	0.0253 (13)	0.0243 (13)	0.0387 (16)	0.0033 (10)	−0.0082 (11)	−0.0033 (12)
N103	0.0167 (12)	0.0252 (14)	0.0335 (15)	0.0006 (10)	0.0018 (11)	−0.0004 (12)
O102	0.0196 (10)	0.0249 (11)	0.0370 (12)	0.0015 (8)	−0.0035 (9)	−0.0004 (10)
C116	0.0258 (16)	0.0304 (18)	0.0415 (19)	0.0009 (13)	−0.0038 (14)	0.0018 (15)
C128	0.0205 (14)	0.0276 (16)	0.0403 (17)	0.0005 (12)	0.0012 (12)	−0.0038 (13)
C110	0.0227 (16)	0.042 (2)	0.0371 (19)	0.0007 (14)	0.0033 (14)	0.0016 (16)
C122	0.0219 (15)	0.0226 (16)	0.0334 (17)	0.0000 (12)	−0.0004 (13)	−0.0030 (14)
C107	0.0315 (17)	0.041 (2)	0.0326 (18)	0.0204 (15)	−0.0063 (14)	−0.0057 (16)
C125	0.0208 (15)	0.0357 (19)	0.052 (2)	−0.0039 (13)	0.0038 (14)	−0.0126 (17)
C121	0.0267 (17)	0.032 (2)	0.037 (2)	0.0015 (14)	0.0043 (14)	−0.0106 (16)
C118	0.0213 (15)	0.0269 (16)	0.0258 (16)	0.0031 (12)	0.0046 (13)	−0.0007 (13)
C111	0.0301 (18)	0.044 (2)	0.053 (2)	−0.0066 (16)	0.0078 (17)	0.0118 (19)
C109	0.0299 (17)	0.0240 (17)	0.0334 (18)	0.0102 (13)	−0.0109 (14)	−0.0072 (14)
C120	0.0206 (15)	0.040 (2)	0.045 (2)	0.0000 (14)	0.0039 (14)	−0.0122 (17)
C114	0.0279 (16)	0.0340 (19)	0.0377 (19)	0.0061 (14)	−0.0001 (14)	0.0048 (16)
C117	0.0276 (15)	0.0256 (17)	0.0300 (16)	0.0041 (13)	0.0012 (13)	−0.0007 (14)
C129	0.0192 (15)	0.0328 (18)	0.053 (2)	0.0029 (13)	−0.0028 (15)	−0.0011 (17)
C119	0.0221 (15)	0.0333 (19)	0.0368 (19)	−0.0022 (13)	0.0018 (14)	−0.0028 (15)
C134	0.0251 (16)	0.044 (2)	0.0375 (18)	−0.0005 (15)	−0.0014 (14)	0.0034 (16)
C127	0.0203 (14)	0.0277 (17)	0.0274 (17)	−0.0025 (13)	0.0007 (12)	−0.0033 (14)
C135	0.0213 (14)	0.0269 (18)	0.0328 (17)	0.0000 (13)	−0.0055 (13)	−0.0013 (14)
C131	0.0293 (16)	0.0392 (19)	0.0346 (19)	0.0025 (14)	−0.0048 (14)	0.0047 (16)
C112	0.0347 (19)	0.034 (2)	0.048 (2)	0.0044 (15)	0.0082 (16)	0.0074 (17)
C115	0.0284 (16)	0.038 (2)	0.048 (2)	0.0093 (15)	−0.0040 (15)	0.0039 (17)
C101	0.0267 (16)	0.0250 (16)	0.050 (2)	0.0008 (13)	−0.0088 (14)	−0.0022 (15)
C113	0.0319 (17)	0.0268 (18)	0.0302 (18)	0.0027 (14)	0.0051 (14)	−0.0002 (15)
C102	0.0318 (17)	0.0267 (18)	0.070 (3)	0.0012 (14)	−0.0184 (18)	−0.0057 (18)
C104	0.0320 (17)	0.0313 (18)	0.057 (2)	0.0131 (16)	−0.0177 (17)	−0.0137 (18)
C136	0.0231 (15)	0.0304 (18)	0.0346 (18)	−0.0037 (13)	−0.0023 (13)	−0.0026 (15)
C14B	0.0279 (16)	0.052 (2)	0.043 (2)	0.0004 (15)	0.0011 (15)	0.0115 (18)
C133	0.041 (2)	0.060 (3)	0.039 (2)	0.008 (2)	0.0059 (19)	0.019 (2)
C126	0.0219 (14)	0.0234 (16)	0.0363 (18)	−0.0006 (12)	0.0017 (13)	−0.0039 (14)
C12A	0.0377 (19)	0.039 (2)	0.052 (2)	0.0090 (16)	−0.0012 (17)	0.0084 (18)
C124	0.0273 (17)	0.038 (2)	0.063 (2)	−0.0136 (15)	−0.0015 (16)	−0.0142 (19)
C108	0.0289 (16)	0.0291 (17)	0.0324 (17)	0.0119 (13)	−0.0084 (14)	−0.0044 (15)
C103	0.0329 (19)	0.034 (2)	0.071 (3)	0.0071 (15)	−0.0234 (19)	−0.0204 (19)
C13A	0.0358 (19)	0.0308 (18)	0.061 (2)	−0.0058 (15)	−0.0002 (17)	−0.0134 (18)
C106	0.041 (2)	0.062 (3)	0.037 (2)	0.029 (2)	−0.0102 (16)	−0.0189 (19)
C11B	0.0334 (18)	0.056 (2)	0.0352 (19)	0.0199 (17)	−0.0002 (15)	−0.0069 (18)
C105	0.044 (2)	0.054 (3)	0.054 (3)	0.0193 (19)	−0.020 (2)	−0.031 (2)
C13B	0.0236 (17)	0.056 (2)	0.093 (3)	−0.0054 (16)	−0.0032 (19)	−0.030 (2)
C12B	0.0256 (16)	0.043 (2)	0.060 (2)	−0.0029 (15)	−0.0115 (16)	0.0133 (19)
C14A	0.056 (3)	0.089 (4)	0.061 (3)	0.032 (2)	0.006 (2)	0.033 (3)
C130	0.0277 (16)	0.040 (2)	0.045 (2)	0.0102 (14)	−0.0068 (15)	0.0014 (17)
C123	0.0296 (16)	0.0275 (18)	0.041 (2)	−0.0038 (14)	0.0031 (14)	−0.0047 (15)
C132	0.041 (2)	0.062 (3)	0.046 (2)	0.0135 (19)	0.0007 (18)	0.017 (2)
C11A	0.056 (3)	0.092 (4)	0.089 (4)	0.020 (3)	−0.021 (3)	−0.064 (3)
N201	0.0424 (18)	0.081 (2)	0.098 (3)	0.0171 (18)	0.001 (2)	0.007 (3)
C202	0.041 (2)	0.080 (3)	0.084 (3)	0.003 (2)	−0.001 (2)	0.010 (3)
C203	0.094 (5)	0.185 (8)	0.154 (7)	0.070 (5)	−0.007 (5)	−0.073 (6)
C201	0.049 (3)	0.090 (4)	0.138 (5)	0.010 (3)	0.004 (3)	0.013 (4)
O201	0.0446 (19)	0.112 (3)	0.131 (4)	0.0012 (19)	0.018 (2)	0.028 (3)
N31	0.285 (12)	0.108 (10)	0.034 (7)	0.057 (10)	0.031 (9)	−0.024 (9)
C31A	0.296 (17)	0.070 (15)	0.055 (11)	−0.019 (14)	0.002 (15)	0.018 (12)
C31B	0.310 (19)	0.106 (14)	0.058 (13)	0.036 (16)	−0.054 (15)	−0.004 (12)
C31C	0.301 (14)	0.100 (11)	0.052 (10)	0.057 (11)	0.030 (12)	0.010 (11)
O31	0.293 (19)	0.149 (12)	0.064 (7)	0.061 (12)	0.002 (15)	0.001 (10)
N32	0.31 (2)	0.136 (15)	0.047 (9)	0.014 (16)	0.053 (16)	0.010 (12)
C32B	0.34 (2)	0.13 (2)	0.045 (13)	−0.03 (2)	0.010 (19)	−0.013 (18)
C32C	0.38 (3)	0.095 (15)	0.032 (11)	0.051 (19)	0.061 (15)	0.000 (10)
C32A	0.35 (2)	0.17 (2)	0.071 (15)	0.027 (19)	0.043 (19)	0.029 (15)
O32	0.39 (3)	0.26 (3)	0.061 (11)	0.14 (2)	−0.011 (18)	0.060 (16)
N33	0.261 (11)	0.078 (8)	0.043 (7)	0.057 (9)	0.005 (8)	−0.020 (7)
C33A	0.243 (15)	0.049 (12)	0.043 (10)	−0.032 (11)	0.019 (12)	−0.018 (9)
C33C	0.307 (17)	0.030 (7)	0.029 (7)	0.007 (9)	−0.020 (10)	−0.005 (6)
C33B	0.267 (14)	0.083 (9)	0.040 (7)	0.059 (11)	−0.025 (11)	−0.009 (9)
O33	0.236 (15)	0.097 (8)	0.094 (10)	0.040 (11)	−0.001 (10)	0.003 (7)

### Geometric parameters (Å, º)

**Table:**

Zr01—O103	2.093 (2)	C12A—H12A	0.96
Zr01—O102	2.100 (2)	C12A—H12B	0.96
Zr01—O101	2.106 (2)	C12A—H12C	0.96
Zr01—O104	2.118 (2)	C124—C123	1.360 (4)
Zr01—N104	2.399 (2)	C124—H124	0.93
Zr01—N103	2.401 (3)	C103—H103	0.93
Zr01—N101	2.435 (3)	C13A—C123	1.505 (5)
Zr01—N102	2.438 (3)	C13A—H13A	0.96
N102—C110	1.310 (4)	C13A—H13B	0.96
N102—C118	1.367 (4)	C13A—H13C	0.96
O101—C108	1.325 (4)	C106—C105	1.361 (6)
O104—C135	1.345 (4)	C106—H106	0.93
N104—C128	1.329 (4)	C11B—H11A	0.96
N104—C136	1.365 (4)	C11B—H11B	0.96
O103—C126	1.340 (4)	C11B—H11C	0.96
N101—C101	1.330 (4)	C105—C11A	1.506 (6)
N101—C109	1.374 (4)	C13B—H13D	0.96
N103—C119	1.314 (4)	C13B—H13E	0.96
N103—C127	1.365 (4)	C13B—H13F	0.96
O102—C117	1.343 (4)	C12B—H12D	0.96
C116—C117	1.380 (4)	C12B—H12E	0.96
C116—C115	1.413 (5)	C12B—H12F	0.96
C116—C12B	1.510 (4)	C14A—C132	1.531 (5)
C128—C129	1.408 (4)	C14A—H14D	0.96
C128—H128	0.93	C14A—H14E	0.96
C110—C111	1.407 (5)	C14A—H14F	0.96
C110—H110	0.93	C130—H130	0.93
C122—C127	1.412 (4)	C11A—H11D	0.96
C122—C121	1.416 (4)	C11A—H11E	0.96
C122—C123	1.421 (4)	C11A—H11F	0.96
C107—C108	1.395 (5)	N201—C201	1.307 (7)
C107—C106	1.422 (5)	N201—C203	1.422 (8)
C107—C11B	1.486 (5)	N201—C202	1.461 (6)
C125—C126	1.373 (4)	C202—H20A	0.96
C125—C124	1.418 (5)	C202—H20B	0.96
C125—C13B	1.508 (4)	C202—H20C	0.96
C121—C120	1.361 (4)	C203—H20D	0.96
C121—H121	0.93	C203—H20E	0.96
C118—C117	1.412 (4)	C203—H20F	0.96
C118—C113	1.424 (5)	C201—O201	1.169 (7)
C111—C112	1.377 (5)	C201—H201	0.93
C111—H111	0.93	N31—C31B	1.341 (17)
C109—C104	1.408 (5)	N31—C31C	1.387 (17)
C109—C108	1.419 (5)	N31—C31A	1.430 (16)
C120—C119	1.410 (5)	C31A—H31A	0.96
C120—H120	0.93	C31A—H31B	0.96
C114—C115	1.372 (4)	C31A—H31C	0.96
C114—C113	1.424 (4)	C31B—H31D	0.96
C114—C12A	1.509 (5)	C31B—H31E	0.96
C129—C130	1.356 (5)	C31B—H31F	0.96
C129—H129	0.93	C31C—O31	1.29 (2)
C119—H119	0.93	C31C—H31J	0.93
C134—C135	1.381 (5)	N32—C32C	1.338 (17)
C134—C133	1.417 (5)	N32—C32A	1.359 (18)
C134—C14B	1.497 (4)	N32—C32B	1.422 (18)
C127—C126	1.417 (4)	C32B—H32A	0.96
C135—C136	1.419 (4)	C32B—H32B	0.96
C131—C132	1.405 (5)	C32B—H32C	0.96
C131—C136	1.413 (4)	C32C—H32D	0.96
C131—C130	1.421 (4)	C32C—H32E	0.96
C112—C113	1.406 (5)	C32C—H32F	0.96
C112—H112	0.93	C32A—O32	1.27 (2)
C115—H115	0.93	C32A—H32J	0.93
C101—C102	1.406 (5)	N33—C33B	1.343 (16)
C101—H101	0.93	N33—C33C	1.392 (15)
C102—C103	1.375 (6)	N33—C33A	1.418 (15)
C102—H102	0.93	C33A—H33A	0.96
C104—C103	1.421 (6)	C33A—H33B	0.96
C104—C105	1.428 (6)	C33A—H33C	0.96
C14B—H14A	0.96	C33C—H33D	0.96
C14B—H14B	0.96	C33C—H33E	0.96
C14B—H14C	0.96	C33C—H33F	0.96
C133—C132	1.374 (5)	C33B—O33	1.28 (2)
C133—H133	0.93	C33B—H33J	0.93
			
O103—Zr01—O102	141.21 (8)	H12A—C12A—H12B	109.5
O103—Zr01—O101	87.00 (8)	C114—C12A—H12C	109.5
O102—Zr01—O101	103.47 (9)	H12A—C12A—H12C	109.5
O103—Zr01—O104	106.07 (8)	H12B—C12A—H12C	109.5
O102—Zr01—O104	89.13 (8)	C123—C124—C125	124.9 (3)
O101—Zr01—O104	141.02 (8)	C123—C124—H124	117.5
O103—Zr01—N104	74.36 (8)	C125—C124—H124	117.5
O102—Zr01—N104	143.99 (8)	O101—C108—C107	122.7 (3)
O101—Zr01—N104	78.77 (9)	O101—C108—C109	118.3 (3)
O104—Zr01—N104	70.22 (8)	C107—C108—C109	118.9 (3)
O103—Zr01—N103	70.55 (8)	C102—C103—C104	120.3 (3)
O102—Zr01—N103	79.49 (8)	C102—C103—H103	119.9
O101—Zr01—N103	142.52 (8)	C104—C103—H103	119.9
O104—Zr01—N103	75.58 (8)	C123—C13A—H13A	109.5
N104—Zr01—N103	120.68 (9)	C123—C13A—H13B	109.5
O103—Zr01—N101	143.46 (8)	H13A—C13A—H13B	109.5
O102—Zr01—N101	73.70 (8)	C123—C13A—H13C	109.5
O101—Zr01—N101	70.11 (9)	H13A—C13A—H13C	109.5
O104—Zr01—N101	78.70 (9)	H13B—C13A—H13C	109.5
N104—Zr01—N101	73.50 (8)	C105—C106—C107	125.9 (4)
N103—Zr01—N101	142.84 (8)	C105—C106—H106	117.1
O103—Zr01—N102	77.60 (8)	C107—C106—H106	117.1
O102—Zr01—N102	69.70 (8)	C107—C11B—H11A	109.5
O101—Zr01—N102	74.80 (8)	C107—C11B—H11B	109.5
O104—Zr01—N102	143.28 (8)	H11A—C11B—H11B	109.5
N104—Zr01—N102	142.11 (8)	C107—C11B—H11C	109.5
N103—Zr01—N102	71.36 (8)	H11A—C11B—H11C	109.5
N101—Zr01—N102	120.46 (9)	H11B—C11B—H11C	109.5
C110—N102—C118	119.1 (3)	C106—C105—C104	117.3 (4)
C110—N102—Zr01	128.5 (2)	C106—C105—C11A	122.3 (4)
C118—N102—Zr01	112.45 (19)	C104—C105—C11A	120.3 (4)
C108—O101—Zr01	124.0 (2)	C125—C13B—H13D	109.5
C135—O104—Zr01	123.64 (18)	C125—C13B—H13E	109.5
C128—N104—C136	118.2 (3)	H13D—C13B—H13E	109.5
C128—N104—Zr01	127.7 (2)	C125—C13B—H13F	109.5
C136—N104—Zr01	114.05 (18)	H13D—C13B—H13F	109.5
C126—O103—Zr01	123.28 (17)	H13E—C13B—H13F	109.5
C101—N101—C109	119.0 (3)	C116—C12B—H12D	109.5
C101—N101—Zr01	128.1 (2)	C116—C12B—H12E	109.5
C109—N101—Zr01	112.3 (2)	H12D—C12B—H12E	109.5
C119—N103—C127	118.8 (3)	C116—C12B—H12F	109.5
C119—N103—Zr01	128.3 (2)	H12D—C12B—H12F	109.5
C127—N103—Zr01	112.86 (19)	H12E—C12B—H12F	109.5
C117—O102—Zr01	124.72 (18)	C132—C14A—H14D	109.5
C117—C116—C115	118.1 (3)	C132—C14A—H14E	109.5
C117—C116—C12B	120.9 (3)	H14D—C14A—H14E	109.5
C115—C116—C12B	120.9 (3)	C132—C14A—H14F	109.5
N104—C128—C129	122.6 (3)	H14D—C14A—H14F	109.5
N104—C128—H128	118.7	H14E—C14A—H14F	109.5
C129—C128—H128	118.7	C129—C130—C131	120.3 (3)
N102—C110—C111	122.7 (3)	C129—C130—H130	119.9
N102—C110—H110	118.7	C131—C130—H130	119.9
C111—C110—H110	118.7	C124—C123—C122	117.3 (3)
C127—C122—C121	116.4 (3)	C124—C123—C13A	122.5 (3)
C127—C122—C123	118.5 (3)	C122—C123—C13A	120.1 (3)
C121—C122—C123	125.1 (3)	C133—C132—C131	117.6 (3)
C108—C107—C106	116.8 (3)	C133—C132—C14A	121.8 (4)
C108—C107—C11B	119.8 (3)	C131—C132—C14A	120.6 (3)
C106—C107—C11B	123.4 (3)	C105—C11A—H11D	109.5
C126—C125—C124	118.4 (3)	C105—C11A—H11E	109.5
C126—C125—C13B	120.1 (3)	H11D—C11A—H11E	109.5
C124—C125—C13B	121.4 (3)	C105—C11A—H11F	109.5
C120—C121—C122	120.3 (3)	H11D—C11A—H11F	109.5
C120—C121—H121	119.8	H11E—C11A—H11F	109.5
C122—C121—H121	119.8	C201—N201—C203	126.3 (6)
N102—C118—C117	115.5 (3)	C201—N201—C202	118.9 (5)
N102—C118—C113	122.5 (3)	C203—N201—C202	114.9 (5)
C117—C118—C113	122.0 (3)	N201—C202—H20A	109.5
C112—C111—C110	119.0 (3)	N201—C202—H20B	109.5
C112—C111—H111	120.5	H20A—C202—H20B	109.5
C110—C111—H111	120.5	N201—C202—H20C	109.5
N101—C109—C104	122.8 (3)	H20A—C202—H20C	109.5
N101—C109—C108	114.6 (3)	H20B—C202—H20C	109.5
C104—C109—C108	122.5 (3)	N201—C203—H20D	109.5
C121—C120—C119	119.0 (3)	N201—C203—H20E	109.5
C121—C120—H120	120.5	H20D—C203—H20E	109.5
C119—C120—H120	120.5	N201—C203—H20F	109.5
C115—C114—C113	116.5 (3)	H20D—C203—H20F	109.5
C115—C114—C12A	122.9 (3)	H20E—C203—H20F	109.5
C113—C114—C12A	120.5 (3)	O201—C201—N201	132.4 (7)
O102—C117—C116	124.3 (3)	O201—C201—H201	113.8
O102—C117—C118	116.9 (3)	N201—C201—H201	113.8
C116—C117—C118	118.8 (3)	C31B—N31—C31C	129 (2)
C130—C129—C128	119.5 (3)	C31B—N31—C31A	118.4 (19)
C130—C129—H129	120.2	C31C—N31—C31A	113.1 (16)
C128—C129—H129	120.2	N31—C31A—H31A	109.5
N103—C119—C120	122.7 (3)	N31—C31A—H31B	109.5
N103—C119—H119	118.6	H31A—C31A—H31B	109.5
C120—C119—H119	118.6	N31—C31A—H31C	109.5
C135—C134—C133	117.8 (3)	H31A—C31A—H31C	109.5
C135—C134—C14B	121.3 (3)	H31B—C31A—H31C	109.5
C133—C134—C14B	120.9 (3)	N31—C31B—H31D	109.5
N103—C127—C122	122.7 (3)	N31—C31B—H31E	109.5
N103—C127—C126	114.9 (3)	H31D—C31B—H31E	109.5
C122—C127—C126	122.4 (3)	N31—C31B—H31F	109.5
O104—C135—C134	124.0 (3)	H31D—C31B—H31F	109.5
O104—C135—C136	117.2 (3)	H31E—C31B—H31F	109.5
C134—C135—C136	118.8 (3)	O31—C31C—N31	106.0 (18)
C132—C131—C136	119.0 (3)	O31—C31C—H31J	127
C132—C131—C130	124.7 (3)	N31—C31C—H31J	127
C136—C131—C130	116.3 (3)	C32C—N32—C32A	118.3 (18)
C111—C112—C113	120.4 (4)	C32C—N32—C32B	117.8 (19)
C111—C112—H112	119.8	C32A—N32—C32B	123.9 (19)
C113—C112—H112	119.8	N32—C32B—H32A	109.5
C114—C115—C116	125.5 (3)	N32—C32B—H32B	109.5
C114—C115—H115	117.2	H32A—C32B—H32B	109.5
C116—C115—H115	117.2	N32—C32B—H32C	109.5
N101—C101—C102	122.0 (3)	H32A—C32B—H32C	109.5
N101—C101—H101	119	H32B—C32B—H32C	109.5
C102—C101—H101	119	N32—C32C—H32D	109.5
C112—C113—C114	124.7 (3)	N32—C32C—H32E	109.5
C112—C113—C118	116.3 (3)	H32D—C32C—H32E	109.5
C114—C113—C118	118.9 (3)	N32—C32C—H32F	109.5
C103—C102—C101	119.5 (3)	H32D—C32C—H32F	109.5
C103—C102—H102	120.3	H32E—C32C—H32F	109.5
C101—C102—H102	120.3	O32—C32A—N32	121 (3)
C109—C104—C103	116.4 (3)	O32—C32A—H32J	119.4
C109—C104—C105	118.5 (4)	N32—C32A—H32J	119.4
C103—C104—C105	125.1 (3)	C33B—N33—C33C	126.8 (17)
N104—C136—C131	123.2 (3)	C33B—N33—C33A	124.9 (17)
N104—C136—C135	114.8 (3)	C33C—N33—C33A	107.1 (14)
C131—C136—C135	122.0 (3)	N33—C33A—H33A	109.5
C134—C14B—H14A	109.5	N33—C33A—H33B	109.5
C134—C14B—H14B	109.5	H33A—C33A—H33B	109.5
H14A—C14B—H14B	109.5	N33—C33A—H33C	109.5
C134—C14B—H14C	109.5	H33A—C33A—H33C	109.5
H14A—C14B—H14C	109.5	H33B—C33A—H33C	109.5
H14B—C14B—H14C	109.5	N33—C33C—H33D	109.5
C132—C133—C134	124.8 (4)	N33—C33C—H33E	109.5
C132—C133—H133	117.6	H33D—C33C—H33E	109.5
C134—C133—H133	117.6	N33—C33C—H33F	109.5
O103—C126—C125	124.4 (3)	H33D—C33C—H33F	109.5
O103—C126—C127	117.3 (2)	H33E—C33C—H33F	109.5
C125—C126—C127	118.3 (3)	O33—C33B—N33	122 (2)
C114—C12A—H12A	109.5	O33—C33B—H33J	119.1
C114—C12A—H12B	109.5	N33—C33B—H33J	119.1

### Hydrogen-bond geometry (Å, º)

**Table:**

*D*—H···*A*	*D*—H	H···*A*	*D*···*A*	*D*—H···*A*
C14*A*—H14*E*···O201^i^	0.96	2.43	3.358 (7)	161

Symmetry code: (i) −*x*+3/2, *y*+1/2, *z*−1/2.
